# Social Class-Based Discrimination and Psychological Symptoms Among Socioeconomically Disadvantaged College Students: The Moderated Mediation Role of Stress Mindset and Rumination

**DOI:** 10.3389/fpsyt.2022.858951

**Published:** 2022-06-06

**Authors:** Jia Wu, Qianfeng Li, Qinglu Wu, Qiaoling Li

**Affiliations:** ^1^Student Affairs Office, Guangzhou City University of Technology, Guangzhou, China; ^2^Institute of Analytical Psychology, City University of Macau, Macau, Macao SAR, China; ^3^Institute of Advanced Studies in Humanities and Social Sciences, Beijing Normal University, Zhuhai, China; ^4^School of Psychology, Henan University, Kaifeng, China

**Keywords:** perceived social class-based discrimination, rumination, stress-is-enhancing mindset, psychological symptoms, socioeconomically disadvantaged college students

## Abstract

Discrimination as a crucial stressor damages the mental health of socioeconomically disadvantaged individuals through increased ruminative thinking. A “stress-is-enhancing” mindset may protect the mental health of socioeconomically disadvantaged individuals under the pressures of perceived discrimination and rumination. This study examined the mediating role of rumination and the moderating role of stress mindset in the relationship between perceived discrimination and psychological symptoms among socioeconomically disadvantaged college students. A total of 919 socioeconomically disadvantaged undergraduate students (48.4% female, ages 17–25) were recruited. The results indicated that perceived discrimination was positively associated with psychological symptoms among socioeconomically disadvantaged undergraduate students through rumination (*B* = 0.11, boot SE = 0.01, boot 95% CIs = [0.08, 0.13]). Importantly, stress mindset moderated the indirect association between perceived discrimination and psychological distress through rumination (*B* = −0.18, boot SE = 0.08, boot 95% CIs = [−0.32, −0.03]). Specifically, compared with individuals with low levels of the stress-is-enhancing mindset, the indirect effect of perceived discrimination on psychological distress through rumination was weaker among individuals with high levels of the stress-is-enhancing mindset. The findings provide support for future intervention practice to promote a stress-is-enhancing mindset to protect the mental health of socioeconomically disadvantaged college students under the pressures of perceived discrimination and rumination.

## Introduction

College students from economically disadvantaged families are vulnerable to the risk of social exclusion and poor life situations, such as low possibilities of completing a college course or earning a degree and unfairness in accessing employment opportunities (1, 2). In China, around 20% of college students were supported by various national financial aids per year due to their economically disadvantaged family’s economic status (3). Because they are either suffering from chronic poverty or have been affected by a serious natural disaster, their family income is insufficient to cover their tuition fees and the other costs involved in studying at college. Although the Chinese government has issued a series of financial assistance policies to help these students completing college courses and earning a degree, their health and wellbeing is still significantly threatened by widespread social class-based discrimination (4).

As an important stressor for socioeconomically disadvantaged populations, perceived social class discrimination represents the negative effects of poverty on their mental health (5). Perceived discrimination refers to individuals’ subjective perceptions of a devalued and threatened social identity due to experiences of mistreatment in the course of their interactions with others in the society (6). Perceived social class-based discrimination commonly occurs among populations living in poverty or in a socioeconomically disadvantaged situation caused by serious natural disaster, chronic disease of family members, and other economic challenges.

### Perceived Discrimination and Psychological Symptoms

Perceived discrimination is a significant risk factor for the wellbeing of college students who are from socioeconomically disadvantaged families (4, 7). These students are likely to have less access to useful resources (e.g., adequate financial support) to facilitate their academic achievement before graduating (8) and are more likely to experience unfair treatment (e.g., a lack of fair employment opportunities) and relatively poor life situations (9). Studies have found that perceived social class discrimination is associated with symptoms of depression and anxiety among college students from socioeconomically disadvantaged families (10, 11). The response styles theory indicates that paying repetitive and passive concern to the causes and results of stress events and situations may lead to psychological symptoms (12). This model of responding to stress is called rumination. It has been widely found that rumination has a negative effect on wellbeing; for example, Liao et al. (13) found that ruminative thoughts was a key mediator in the relationship between discrimination experience and wellbeing among minority sexualities. However, it remains unclear whether this mechanism operates in the relationship between perceived social class discrimination and psychological symptoms among individuals in socioeconomic disadvantaged situations.

It is noteworthy that some individuals are less likely to report severe psychological symptoms despite the experience of discrimination (14). For example, Li et al. (15) found that despite being affected by parental HIV and discrimination, some children did not report clinically significant mental health problems. Moreover, stress does not always bring negative consequences to individuals. If individuals believe that stress is an opportunity that can be utilized for their personal development, it can improve their performance and stimulate effective coping (16). Implicit theory indicates that individual’s understanding of and response to complex information and situations depends on a simplifying system (i.e., mindset) of which they are unaware (16, 17). Stress mindset refers to the extent to which an individual believes that stress has an enhancing or debilitating effect on learning, performance, health, and wellbeing (16). Specifically, individuals who have the mindset that stress is enhancing are less likely to interpret a stressful event or situation as always having a debilitating influence on their performance and wellbeing, and this can diminish the negative effects of adversity (16). However, there has been limited research into the protective effect of a stress-is-enhancing mindset on mental health and wellbeing among population from socioeconomically disadvantaged families. Simultaneously investigating the roles of rumination and stress mindset in the relationship between perceived social class discrimination and psychological symptoms may enhance our understanding of why some socioeconomically disadvantaged population adapts better than others in a discriminatory situation.

### The Mediating Role of Rumination

According to the response styles theory, rumination is one major maladaptive cognitive response to individuals’ unfavorable experiences, and it involves repetitive and passive attention to disadvantaged settings and related problems (12). Perceived threats and uncontrollable negative events increase the rumination activity (18). Social class discrimination experiences, such as being disrespected in the course of interpersonal communications and being treated unfairly by others due to low socioeconomic status (SES), can lead individuals to perceive a discrepancy between the prevailing and desired status. Thereafter, they are likely to think repetitively over the deeper implications of this discrepancy and experience negative affect when the discrepancy persists (19).

Rumination leads to increased negative thoughts and moods and poor problem solving, which may further exacerbate psychological symptoms, such as depression and anxiety (18). Individuals with increased ruminative thoughts may perceive more hopelessness and overgeneralize that all their efforts are fruitless in a disadvantaged situation (19). Rumination partially mediates the association between stressful life events (e.g., discrimination) and psychological symptoms (18, 20). Accordingly, perceived social class discrimination may be positively associated with increased rumination, which could in turn be positively related to psychological symptoms among socioeconomically disadvantaged populations.

### The Buffering Role of Stress-Is-Enhancing Mindset

Although psychological symptoms arising from perceived social class discrimination and rumination have potentially debilitating impacts on the mental health of individuals (6), certain understandings of the nature of stress may help individuals to alleviate the negative effect of these negative experiences (16). The acquired mindset about stress is considered as a cognitive frame that influences individuals’ understanding of complex and conflicting situations and triggers certain reactions (16, 17). According to stress mindset theory, individuals holding the “stress-is-enhancing” mindset are more likely to focus on the positive and enriching consequences (e.g., wellbeing, learning, and thriving) of stress and to believe stress could be utilized positively. Conversely, individuals holding the “stress-is-debilitating” mindset are more likely to focus on the negative and detrimental consequences (e.g., psychological symptoms) of stress and to believe that stress should be avoided (16).

Empirical studies have demonstrated that the effects of stress on health-related outcomes may differ based on various stress mindsets (16, 21, 22). Individuals with a stress-is-enhancing mindset are more likely to focus on the positive effects of stress (16) and accept the prevailing situation and its consequences. Acceptance rather than avoidance of stress experiences may help individuals minimize the negative effects of stressful events and ruminative thoughts on mental health [(23); refer also to the review by Nolen-Hoeksema et al. (19)]. These individuals are more inclined to consider the likelihood of positive consequences despite experiencing adversity, for example, by believing that their capacity to cope with stress will be improved (24). Therefore, college students from socioeconomic disadvantaged families with the stress-is-enhancing mindset might be more likely to focus on the positive aspects of a stress rather than engage in repetitive and passive attention to its negative effects. In contrast, holding the stress-is-debilitating mindset might increase the negative effects of stress on those individuals’ health outcomes, with more negative psychological symptoms reported (16). Therefore, the stress-is-enhancing mindset could minimize the negative effects of stress on individuals’ mental health when the stimuli are persistent or do not diminish within a short period (16).

Increasing empirical evidence suggests that the stress-is-enhancing mindset protects the mental health, physical health, and academic performance of individuals experiencing stressful events, such as discrimination (21, 22, 25). Individuals with a stress-is-enhancing mindset reported fewer depressive symptoms when facing stressful life events (26). However, limited studies explored whether the association between perceived social class discrimination and psychological symptoms is weaker among socioeconomic disadvantaged college students with a stress-is-enhancing mindset than those students with a stress-is-debilitating mindset. Inspired by stress mindset theory, we expected the negative effect of discrimination experiences on mental health status to decline among those socioeconomic disadvantaged college students with a stress-is-enhancing mindset.

Furthermore, the negative effect of rumination on individuals’ mental health may be buffered by the stress-is-enhancing mindset. When individuals have a stress-is-enhancing mindset, they are more likely to interpret the prevailing stress as potentially improving their resilience, leading them to thrive in the stress reappraising process and accept the situation (19). Therefore, it is critical to investigate if the association between rumination and psychological symptoms in individuals under the pressure of discrimination experiences is weaker among those with a stress-is-enhancing mindset and stronger among those with a stress-is-debilitating mindset.

### This Study

**FIGURE 1:**
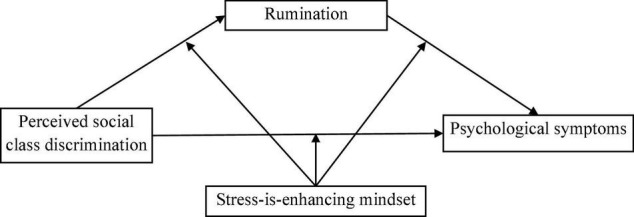
The proposed moderated mediation model. Controlling for age, gender, family income, parents’ education attainment, and subjective socioeconomical status.

Our previous study found that perceived social class discrimination negatively associated with college students’ wellbeing (7). The potential role of rumination and the buffering effect of stress mindset in the relationship between perceived social class discrimination and psychological symptoms were investigated in this study. We proposed a moderated mediation model to examine the association between perceived social class discrimination and psychological symptoms among socioeconomically disadvantaged college students and the mediating role of rumination and the moderating role of stress mindset in this association (refer to Figure 1). We used the symptoms of depression, anxiety, and stress as indicators of psychological symptoms. The following hypotheses were proposed:

Hypothesis 1 (H1): Perceived social class discrimination is positively associated with psychological symptoms among socioeconomically disadvantaged individuals.

Hypothesis 2 (H2): Perceived social class discrimination is indirectly associated with psychological symptoms through increased rumination. Specifically, perceived social class discrimination is positively associated with rumination, which in turn increases psychological symptoms among college students from socioeconomically disadvantaged families.

Hypothesis 3 (H3): The direct effect of perceived social class discrimination on psychological symptoms is buffered by the stress-is-enhancing mindset. Specifically, the association between perceived social class discrimination and psychological symptoms is weaker among individuals with high levels of the stress-is-enhancing mindset and stronger among individuals with low levels of the stress-is-enhancing mindset.

Hypothesis 4 (H4): The indirect association between perceived social class discrimination and psychological symptoms through rumination is moderated by the stress-is-enhancing mindset. Specifically, the negative association between perceived social class discrimination and rumination is weaker among college students with high levels of the stress-is-enhancing mindset and stronger among those students with low levels of the stress-is-enhancing mindset. Moreover, the negative association between rumination and psychological symptoms is weaker among college students with high levels of the stress-is-enhancing mindset and stronger among those students with low levels of the stress-is-enhancing mindset.

## Materials and Methods

### Participants and Procedure

The sample consisted of 919 (i.e., 447 female and 472 male) undergraduate students with low family SES enrolled in the first, second, or third year of their studies in a university in Guangzhou, China (7). Eligible students were those who applied for financial aid in the last year. The eligibility criteria for this financial aid were (1) average annual per capita household income below the amount stipulated for the application for financial aid at the university and (2) family members suffering from serious disease or experienced major natural disasters and experiencing economic challenges. Undergraduate students in their final year were not recruited due to their imminent graduation at the time. The ages of the participants ranged from 17 to 25 years (*M* = 20.46 years, *SD* = 1.33 years). The convenience sampling method was applied to recruit eligible participants from the eligible students list, and their personal information (e.g., name and family members’ information) was kept strictly confidential. The eligible students’ list was identified by the student affairs office of the university. All participants answered the online questionnaire through Qualtrics (Qualtrics, Provo, UT, United States) after providing individual written consent. The written informed consent was obtained from all the participants. If the student aged < 18 years, the electronic copy of written consent would be obtained from participants’ legal guardian (e.g., parents) through the WeChat (an instant messaging app). The research procedures performed in studies involving human participants were approved by the Institutional Review Board of the Guangzhou City University of Technology.

### Measurements

#### Demographic Variables

The demographic variables were participants’ age, sex, father’s educational level and mother’s educational level, household annual income, and subjective SES (SSES). The participants responded to their parents’ educational attainment by four options (1 = “elementary school and below,” 2 = “junior high school,” 3 = “senior high school,” and 4 = “bachelors and above”). In addition, the SSES was measured by a visual scale with 10-rung ladders (27). The participants responded by ranking their perception of family SES on the ladder relative to other students in the university. The score ranged from 1 to 10, and a higher score indicated higher subjective SES.

#### Perceived Social Class Discrimination

The Perceived Discrimination Scale (28) with six items was used to measure the college students’ perception of discrimination experiences due to their low family SES. The participants responded based on their feelings of discrimination (e.g., “I feel that people treat me differently because of my family social class background”) on a 5-point Likert scale (from 1 = *strongly disagree* to 5 = *strongly agree*). A previous study reported that the scale had adequate consistency and reliability for measuring the perception of discrimination among Chinese college students (29). Higher mean scores were taken to indicate higher perceived social class discrimination (Cronbach’s α = 0.89).

#### Rumination

The ten-item Chinese version of the Short Ruminative Responses Scale (SRRS) [(30); H. (31)] was used to measure participants’ tendencies to engage in ruminative thoughts (e.g., “Think about how passive and unmotivated you felt” and “Think about a recent situation, wishing it had gone better”). The responses were rated on a 4-point scale (from 1 = *never* to 4 = *always*). Higher mean scores were taken as reflecting higher frequencies of ruminative thoughts (Cronbach’s α = 0.88).

#### Stress Mindset

The eight-item Chinese version of the Stress Mindset Measure (16, 26) was used to assess participants’ beliefs about the nature of stress (i.e., stress is enhancing vs. stress is debilitating). Participants responded on a 5-point Likert scale (from 1 = *strongly disagree* to 5 = *strongly agree*). Example items were “The effects of stress are positive and should be utilized” and “Experiencing this stress depletes my health and vitality.” After reversing the negatively worded items, a higher mean score indicated a higher level of the stress-is-enhancing mindset. We used the term “stress-is-enhancing mindset” consistently in the results to avoid confusion, with high levels of the stress-is-enhancing mindset equivalent to low levels of the stress-is-debilitating mindset (Cronbach’s α = 0.76).

#### Psychological Symptoms

Participants’ psychological symptoms were measured by the Chinese version of the Depression Anxiety Stress Scale with 21 items (DASS-21) (32, 33). The participants rated the extent to which they felt certain symptoms (e.g., “I was worried about situations in which I might panic”) on a 4-point scale (from 0 = *did not apply to me at all* to 3 = *applied to me very much*). According to the recommendation of the developers of the scale (32), the sum score was multiplied by 2 to obtain a final score. A higher final score indicated a higher level of psychological symptoms (Cronbach’s α = 0.94).

### Data Analysis

To minimize the common method bias, all participants were informed that there were no correct or incorrect responses for the items in the survey. Moreover, we conducted the Harman’s single-factor test to evaluate whether the common method bias was serious (34). According to an exploratory factor analysis on all key variables, the extracted first component accounted for 26.3% of the total variance. This indicated that the common method bias was not severe in this study.

In the preliminary analysis, Pearson correlation coefficients were used to analyze bivariate correlations among the key variables. Furthermore, the proposed moderated mediation model was examined by the PROCESS macro [model 59; (35)], which tested the direct and indirect effects of perceived social class discrimination on psychological symptoms and the potential moderating role of stress mindset. First, assisted by PROCESS macro, the bootstrapping approach was used as the robust analysis to test the indirect effect of perceived social class discrimination on psychological symptoms through rumination (36). The indirect effects were considered significant if the bootstrapped 95% confidence intervals (boot 95% CIs) did not include zero (37). Second, the conditional indirect effect analysis was used to examine whether the indirect effects of perceived social class discrimination on psychological symptoms at high (*M* + 1*SD*) and low (*M* - 1*SD*) levels of the stress-is-enhancing mindset were significantly different. Third, the simple slope analysis was performed to analyze the nature of the moderation effects.

## Results

### Preliminary Analyses

The participants’ demographic characteristics are presented in Table 1. The results of bivariate correlation (refer to Table 2) showed that perceived social class discrimination was positively associated with rumination and psychological symptoms and was negatively associated with the stress-is-enhancing mindset. The stress-is-enhancing mindset was negatively associated with rumination and psychological symptoms. Rumination was positively associated with psychological symptoms. In addition, SSES was negatively associated with perceived social class discrimination, rumination, and psychological symptoms and was positively associated with the stress-is-enhancing mindset.

**TABLE 1:** Sample demographics.

Variable	Overall *n* (%)	Females *n* (%)	Males *n* (%)
*n*	919 (100%)	447 (48.4%)	472 (51.6%)
**Year in College**			
Freshman	257 (28%)	133 (29.7%)	124 (26.0%)
Sophomore	294 (32%)	112 (25.2%)	182 (38.7%)
Junior	367 (40%)	202 (45.1%)	165 (35.3%)
**Father’s education attainment**			
Elementary school and below	283 (30.8%)	132 (29.5%)	151 (32.0%)
Junior high school	444 (48.3%)	225 (50.3%)	219 (46.4%)
Senior high school	171 (18.6%)	85 (19.1%)	86 (18.2%)
Bachelors and above	21 (2.3%)	5 (1.1%)	16 (3.4%)
**Mother’s education attainment**			
Elementary school and below	466 (50.7%)	225 (50.3%)	241 (51.1%)
Junior high school	359 (39.1%)	178 (39.8%)	181 (38.3%)
Senior high school	85 (9.2%)	44 (9.7%)	50 (8.9%)
Bachelors and above	9 (1.0%)	1(0.2%)	8 (1.7%)
	*M* (*SD*)	*M* (*SD*)	*M* (*SD*)
Age	20.46 (1.33)	20.32 (1.33)	20.58 (1.32)
SSES	5.32 (1.22)	5.21 (1.20)	5.41 (1.24)
PSCD	2.57 (0.77)	2.54 (0.78)	2.59 (0.76)
Rumination	2.13 (0.44)	2.13 (0.43)	2.12 (0.45)
SIEM	3.42 (0.45)	3.39 (0.46)	3.44 (0.45)
Psychological symptoms	66.90 (19.73)	65.60 (17.34)	68.12 (21.67)

*SSES, subjective socioeconomic status; PSCD, perceived social class discrimination; SIEM, stress-is-enhancing mindset.*

**TABLE 2:** Correlations between key variables.

	1	2	3	4	5
1. SSES	−				
2. PSCD	−0.23**	−			
3. Rumination	−0.24**	0.43**	−		
4. SIEM	0.20**	−0.26**	−0.14**	–	
5. Psychological symptoms	−0.26**	0.46**	0.58**	−0.29**	–

*SSES, subjective socioeconomic status; SIEM, stress-is-enhancing mindset.*

**p < 0.01.

### Testing for Proposed Moderated Mediation Model

After controlling for age, sex, family income, parents’ educational level, and SSES, the results showed that perceived social class discrimination was positively associated with psychological symptoms through rumination (direct effect: *B* = 0.12, boot SE = 0.02, boot 95% CIs = [0.09, 0.16]; indirect effect: *B* = 0.11, boot SE = 0.01, boot 95% CIs = [0.08, 0.13]). Moreover, the interaction between perceived social class discrimination and stress mindset had no significant effect on psychological symptoms (*B* = 0.02, boot SE = 0.05, boot 95% CIs = [−0.08, 0.11]), which indicated that the direct association between perceived social class discrimination and psychological symptoms was not moderated by the stress-is-enhancing mindset. Furthermore, the interaction between perceived social class discrimination and the stress-is-enhancing mindset had no significant effect on rumination (*B* = −0.02, boot SE = 0.04, boot 95% CIs = [−0.09, 0.06]), whereas the interaction between rumination and the stress-is-enhancing mindset had a significant effect on psychological symptoms (*B* = −0.18, boot SE = 0.08, boot 95% CIs = [−0.32, −0.03]). This finding indicated that the indirect association between perceived social class discrimination and psychological symptoms through rumination was moderated by the stress-is-enhancing mindset, specifically in the association between rumination and psychological symptoms Table 3.

**TABLE 3:** The moderated mediation model analysis.

Variable	*B*	Boot SE	Bias-corrected boot 95 CIs	
			LLCI	ULCI
**Outcome: rumination**				
Age	–0.01	0.01	–0.03	0.01
Sex (male = 1)	–0.02	0.02	–0.07	0.04
Family income	0.06	0.05	–0.04	0.16
Parents education attainment	0.03	0.02	–0.01	0.08
SSES	−0.05***	0.01	–0.07	–0.03
PSCD	0.23***	0.02	0.18	0.27
SIEM	–0.01	0.03	–0.08	0.05
Perceived discrimination × SIEM	–0.02	0.04	–0.09	0.06
**Outcome: psychological symptoms**				
Age	0.01	0.01	–0.01	0.02
Sex (male = 1)	0.07**	0.02	0.02	0.12
Family income	0.00	0.05	–0.09	0.09
Parents’ education attainment	0.00	0.02	–0.05	0.04
SSES	−0.03**	0.01	–0.05	–0.01
PSCD	0.12***	0.02	0.08	0.16
Rumination	0.48***	0.04	0.40	0.55
SIEM	−0.17***	0.03	–0.23	–0.11
Perceived discrimination × SIEM	0.02	0.05	–0.08	0.11
Rumination × SIEM	−0.18**	0.08	–0.32	–0.03
**Conditional indirect effect analysis**				
Low SIEM (*M* − 1*SD*)	0.13	0.02	0.09	0.17
High SIEM (*M* + 1*SD*)	0.09	0.02	0.06	0.12

*Bootstrap sample size = 5,000. LLCI, lower limit confidence interval; ULCI, upper limit confidence interval; SE, standard error; SSES, subjective socioeconomic status; SIEM, stress-is-enhancing mindset.*

**p < 0.01, ***p < 0.001.

**FIGURE 2:**
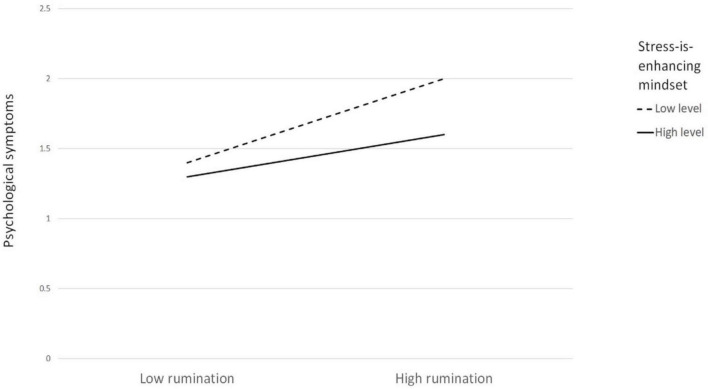
The interaction between rumination and stress-is-enhancing mindset on psychological symptoms.

According to the results of the conditional indirect effect analysis, compared with individuals with low levels of the stress-is-enhancing mindset (i.e., the level of stress mindset at *M* - 1*SD*), the indirect positive effect of perceived social class discrimination on psychological symptoms through rumination was weaker among individuals with high levels of the stress-is-enhancing mindset (i.e., the level of the stress mindset at *M* + 1*SD*). A simple slope analysis was performed Table 3, and the result showed a significant difference between the two conditional indirect effects (contrast = −0.04, boot SE = 0.02, boot 95% CIs = [−0.09, −0.01]). Furthermore, the relationship between rumination and psychological symptoms in the participants with low and high levels of the stress-is-enhancing mindset is plotted in Figure 2. In summary, the results indicate that the direct effects of perceived social class discrimination on psychological symptoms were not moderated by the stress-is-enhancing mindset, whereas the indirect effects of perceived social class discrimination on psychological symptoms through rumination were significantly moderated by the stress-is-enhancing mindset.

## Discussion

This study empirically demonstrated how the stress mindset influences the complex relationships among perceived social class discrimination, rumination, and psychological symptoms among socioeconomically disadvantaged populations. The findings of this study partly support our hypotheses (H3 was not supported, and H4 was partly supported). Consistent with the results of a previous study (18), the findings of this study indicate a positive association between perceived social class discrimination and psychological symptoms among college students from socioeconomically disadvantaged families and a mediating role of rumination in this relationship. Importantly, the finding highlights the stress-is-enhancing mindset as a buffer in the indirect association between perceived social class discrimination and psychological symptoms through rumination, although the stress-is-enhancing mindset may not buffer the direct association between perceived social class discrimination and psychological symptoms.

### Perceived Social Class Discrimination and Psychological Symptoms

The finding indicates that when socioeconomically disadvantaged college students perceive higher discrimination, they report higher psychological symptoms. The finding supports perceived social class discrimination as a key risk factor for the mental health status of socioeconomically disadvantaged population (6, 9). Among this population, financial pressures and limited external and personal resources increase individuals’ awareness of the difference between their family backgrounds and those of their peers (38), which in turn enhances their perception of discrimination and leads to poor psychological wellbeing and negative health outcomes (6, 9). The findings implicate that the education and services of mental health for college youths should focus on reducing the threat of social class discrimination, especially for socioeconomically disadvantaged students.

### The Mediating Role of Rumination

The findings reveal that perceived social class discrimination was positively associated with psychological symptoms through increased rumination, which supported previous findings (13, 18). Rumination is a maladaptive response for individuals facing adversity and stress events (19). When individuals succumb to these negative and repetitive responses to discrimination experiences, they may feel hopeless in adapting to the prevailing adversity and trigger more psychological symptoms. Furthermore, considering the characteristics of intergenerationally transmitted poverty (39, 40), socioeconomically disadvantaged college students may believe that their low family SES is fixed for the long term, and those pessimistic and fatalistic thoughts can lead to rumination and threaten their mental health (41, 42). The future practice could explore the effective approach (e.g., mindfulness training) in decreasing the ruminative thoughts among socioeconomically disadvantaged college students, which may be helpful for diminishing the psychological symptoms of the college student when they suffer from discrimination.

### The Buffering Role of Stress-Is-Enhancing Mindset

Importantly, the findings indicate that a stress-is-enhancing mindset can weaken the effects of the rumination on psychological symptoms among socioeconomically disadvantaged college students when they are exposed to stressful situations (21, 22, 25), particularly when they engage in rumination due to perceived social class discrimination. The benefit of a stress-is-enhancing mindset in buffering the negative effect of stress may be more likely to be realized when individuals evaluate stress as a challenge rather than a threat (43). Researchers have suggested that rumination is a type of a coping strategy involving positive and negative aspects (44). When individuals interpret rumination as a useful coping strategy (45), they may anticipate positive effects of rumination on downstream outcomes, such as resilience. For instance, college students experiencing discrimination due to their low family SES backgrounds might engage in rumination. However, when they believe that rumination does not always have negative consequences, they can reappraise the prevailing stressful situation positively, which may motivate them to focus more on how to adapt and thrive under such environments based on their strengths (e.g., academic ability) rather than perceived weaknesses (e.g., a socioeconomically disadvantaged background), and this will in turn protect their mental health (16). Although perceived discrimination causes rumination and psychological symptoms, holding the belief of the stress-is-enhancing mindset could buffer the negative effect of rumination on mental health among socioeconomically disadvantaged college students. The school health care services could consider developing and providing a stress-is-enhancing mindset intervention to protect student’s mental health.

Moreover, the findings indicate that the moderating role of stress mindset in the association between perceived social class discrimination and rumination is not significant. It is possible that when the perceived social class discrimination is perceived as a key threat directly leading to ruminative thoughts and affecting college students’ mental health (e.g., symptoms of depression and anxiety), the stress-is-enhancing mindset may not protect against repeated negative thoughts and thus the negative effects of discrimination on mental health in highly stressful situations (43). This might explain why the findings of this study did not support the view that a stress-is-enhancing mindset buffers the direct effects of perceived social class discrimination on psychological symptoms in college students. Future studies may examine the buffering effect of the stress-is-enhancing mindset in the relationship between perceived social class discrimination and wellbeing in other populations.

### Limitations

Some limitations of this study should be considered. First, this study is the first to examine the stress mindset of the target population with a large-scale survey and is therefore implemented as a preliminary investigation with data collected at one specific time point and with no experimental manipulation of the natural environment. Although inferences can be made from the findings about the relationships among the variables of interest, the cross-sectional design of the study means that it cannot reveal the causal relationships among these variables. Future studies should apply rigorous study designs to explore whether the negative effects of perceived social class discrimination and rumination can be buffered by the stress-is-enhancing mindset. Second, it remains unclear whether a stress mindset can protect an individual’s mental health status over time under the sustained threat of perceived social class discrimination and rumination. Third, this study recruited participants from university students. The findings may lack generalizability across different areas and cultures. Future studies may examine the protective effect of the stress-is-enhancing mindset on mental health among other socioeconomically disadvantaged population, such as children affected by parental HIV/AIDS.

## Conclusion

The findings of this study suggest that stress mindset could buffer the effects of stress on vulnerable individuals’ mental health (16, 22). The findings facilitate our understanding of how stress-is-enhancing mindsets influence individuals’ capacities to maintain good mental health and resist psychological symptoms despite the pressures of poverty. Moreover, the findings make a theoretical contribution in expanding the understanding of the buffering effect of a stress-is-enhancing mindset in the indirect association between stress and mental health outcomes. In particular, it appears that reducing the negative effects of rumination is important in highly stressful situations when the stress-is-enhancing mindset cannot directly protect individuals’ mental health. This study provides insights in designing future stress-is-enhancing mindset-based interventions for socioeconomically disadvantaged students. Such intervention may help vulnerable individuals to focus on the positive consequences of stress, use it as a strategy to reduce the potential of severe psychological symptoms, and maintain a positive outlook regarding their disadvantaged life situations.

## Data Availability Statement

The original contributions presented in the study are included in the article/supplementary material, further inquiries can be directed to the corresponding authors.

## Ethics Statement

The studies involving human participants were reviewed and approved by the Research Ethics Committee of the Guangzhou City University of Technology. Written informed consent to participate in this study was provided by the participants or their legal guardian/next of kin.

## Author Contributions

JW: designed and executed the study, collected the data, and wrote the manuscript. QFL: designed and executed the study, developed conceptual framework, wrote the manuscript, and critically reviewed the manuscript. QW: developed conceptual framework and critically reviewed the manuscript. QLL: edited and critically reviewed the manuscript. All authors approved the final version of the manuscript for submission.

## Conflict of Interest

The authors declare that the research was conducted in the absence of any commercial or financial relationships that could be construed as a potential conflict of interest.

## Publisher’s Note

All claims expressed in this article are solely those of the authors and do not necessarily represent those of their affiliated organizations, or those of the publisher, the editors and the reviewers. Any product that may be evaluated in this article, or claim that may be made by its manufacturer, is not guaranteed or endorsed by the publisher.
